# Inflammation Mediated Metastasis: Immune Induced Epithelial-To-Mesenchymal Transition in Inflammatory Breast Cancer Cells

**DOI:** 10.1371/journal.pone.0132710

**Published:** 2015-07-24

**Authors:** Evan N. Cohen, Hui Gao, Simone Anfossi, Michal Mego, Neelima G. Reddy, Bisrat Debeb, Antonio Giordano, Sanda Tin, Qiong Wu, Raul J. Garza, Massimo Cristofanilli, Sendurai A. Mani, Denise A. Croix, Naoto T. Ueno, Wendy A. Woodward, Raja Luthra, Savitri Krishnamurthy, James M. Reuben

**Affiliations:** 1 Department of Hematopathology, The University of Texas MD Anderson Cancer Center, Houston, Texas, United States of America; 2 Department of Breast Medical Oncology, The University of Texas MD Anderson Cancer Center, Houston, Texas, United States of America; 3 Department of Pathology, The University of Texas MD Anderson Cancer Center, Houston, Texas, United States of America; 4 Department of Radiation Oncology, The University of Texas MD Anderson Cancer Center, Houston, Texas, United States of America; 5 The Morgan Welch Inflammatory Breast Cancer Research Program and Clinic, The University of Texas MD Anderson Cancer Center, Houston, Texas, United States of America; 6 The University of Texas Graduate School of Biomedical Sciences at Houston, The University of Texas MD Anderson Cancer Center, Houston, Texas, United States of America; 7 National Cancer Institute, Bratislava, Slovak Republic; 8 Medical Oncology, Thomas Jefferson University, Philadelphia, PA, United States of America; 9 Roche Diagnostics, Indianapolis, IN, United States of America; The University of Hong Kong, CHINA

## Abstract

Inflammatory breast cancer (IBC) is the most insidious form of locally advanced breast cancer; about a third of patients have distant metastasis at initial staging. Emerging evidence suggests that host factors in the tumor microenvironment may interact with underlying IBC cells to make them aggressive. It is unknown whether immune cells associated to the IBC microenvironment play a role in this scenario to transiently promote epithelial to mesenchymal transition (EMT) in these cells. We hypothesized that soluble factors secreted by activated immune cells can induce an EMT in IBC and thus promote metastasis. In a pilot study of 16 breast cancer patients, TNF-α production by peripheral blood T cells was correlated with the detection of circulating tumor cells expressing EMT markers. In a variety of IBC model cell lines, soluble factors from activated T cells induced expression of EMT-related genes, including *FN1*, *VIM*, *TGM2*, *ZEB1*. Interestingly, although IBC cells exhibited increased invasion and migration following exposure to immune factors, the expression of E-cadherin (*CDH1*), a cell adhesion molecule, increased uniquely in IBC cell lines but not in non-IBC cell lines. A combination of TNF-α, IL-6, and TGF-β was able to recapitulate EMT induction in IBC, and conditioned media preloaded with neutralizing antibodies against these factors exhibited decreased EMT. These data suggest that release of cytokines by activated immune cells may contribute to the aggressiveness of IBC and highlight these factors as potential target mediators of immune-IBC interaction.

## Introduction

Inflammatory breast cancer (IBC) is the most aggressive form of locally advanced breast cancer, characterized by diffuse erythema and edema of the breast that is often mistaken for mastitis. Yet IBC is not considered a true inflammatory condition; rather inflammation that arises from the characteristic tumor emboli that block the dermal lymphatics [1]. The tumor progresses quickly, often within a few weeks or months, and is often metastatic at diagnosis [2, 3]. This rapid onset of metastasis suggests lymphatic or hematogenous dissemination at an early stage of disease.

Since tumor cells are in regular contact with immune cells trafficking through the lymphatics, the lack of tumor control is suggestive of a compromised immune surveillance system. However, IBC may present with an augmented cellular immune response to tumor antigen and showed that IBC patients have normal delayed-type hypersensitivity reactions to standard recall antigens and breast tumor lysate [4]. The effect of this postulated immune response on tumor cells and their metastatic potential is only beginning to be explored. Recently, it was shown that when factors secreted by the monocytic cell line U937 are added to cultures of the IBC cell line SUM149, the tumor cells develop enhanced migratory and invasive features [5] and increased expression of fibronectin [6]. Activated immune cells are capable of producing factors such as tumor necrosis factor (TNF)-α, interleukin (IL)-6, IL-1β, and transforming growth factor (TGF)-β that induce epithelial-to-mesenchymal transition (EMT). These characteristics suggest that immune cells may induce an EMT in IBC.

EMT is a set of biological processes that occur as epithelial cells lose their sedentary characteristics and gain a motile phenotype. Type I EMT [7], characteristic of cell migration during embryogenesis is characterized by expression of genes such as *SOX2*, *SNAI1*, and *SNAI2* that encode transcription factors that control EMT. Type II EMT, involved in wound repair, tissue regeneration, and fibrosis, is characterized by inflammation, and TGF-β signaling is frequently involved. Neoplastic cells undergoing type III EMT hijack hallmarks of both of these programs, producing a metastatic pathology. EMT, regardless of type, is characterized primarily by a loss of E-cadherin (CDH1) expression mediated by the expression of transcription factors such as SNAI1 [8], SNAI2[9], and ZEB1 [10] that bind directly to E-box regions in the E-cadherin promoter and repress protein expression[11]. *TWIST1* [12], mesenchyme forkhead 1 (*FOXC2*) [13], and tissue transglutaminase (*TGM2*) [14, 15] have also been shown to regulate EMT. We have recently demonstrated that detection of any of these EMT-related transcription factors (EMT-TFs) or *TGM2* in the peripheral blood of breast cancer patients can serve as a surrogate for circulating tumor cells (CTC) in breast cancer patients [16]. Furthermore, the detection of EMT factors in blood was correlated with the detection of CTC with stem-cell phenotypes [17]. Interestingly, although acquisition of EMT characteristics appears to be a necessary step in metastatic progression, IBC tumors are characterized by high levels of the cell adhesion molecule E-cadherin, even in metastatic sites [18, 19] leading some to postulate that E-cadherin may promote tumor progression in this disease [20].

Although cytokines such as TNF-α, TGF-1 [21], IL-6 [22], and IL-1β are capable of inducing EMT in breast cancer cells, the source of these factors has not been studied. We hypothesized that activated immune cells can deliver such factor to the tumor microenvironment. In the current study, soluble factors secreted by healthy donor peripheral blood mononuclear cells (PBMC) were added to cultures of breast cancer cells. Through transcriptional profiling and real-time cell analysis, we found that secreted factors from activated immune cells are capable of inducing EMT in IBC cells. Paradoxically and unique to IBC, the induction of EMT was concomitant with increased E-cadherin expression as characteristically seen in tumor samples.

## Materials and Methods

### Ethics statement

The study has been approved by the Institutional Review Board (IRB) at The University of Texas MD Anderson Cancer Center, and adhered to the tenets of the Declaration of Helsinki. Written informed consent was obtained from each participant prior to sample collection.

### EMT in circulating tumor cells from patients

Blood was collected under IRB-approved protocols LAB08-0199 and LAB08-0079 for the detection of CTC undergoing EMT (EMT-CTC) by real-time reverse transcription-polymerase chain reaction (RT-PCR) following depletion of CD45+ leukocytes [16]. Matched archived PBMCs were stimulated overnight through the T-cell receptor (TCR) with immobilized anti-CD3 and soluble anti-CD28 antibodies and stained for intracellular cytokine production of TNF-α for analysis by flow cytometry [23, 24].

### Cell lines

IBC cell lines were grown in IBC medium in 2D culture, as previously described [25, 26]. Additional breast cancer cell lines are outlined in S1 Table. All cell lines were grown at 37°C in a 5% carbon dioxide humidified atmosphere in standard 2-D culture. SUM149 and SUM190 were obtained from Dr. Stephen Ethier (Kramanos Institute, MI, USA) and are commercially available (Asterand, Detroit, MI). IBC-3 cells were provided courtesy of Dr. Wendy Woodward[26]. KPL4 was provided courtesy of Dr. Junichi Kurebayashi (Kawasaki Medical School, Kurashiki, Japan) [27].

### Preparation of activated immune cell conditioned medium

Fresh PBMCs were cultured at an initial density of 1 x 10^6^ cells/mL in RPMI 1640 medium supplemented with 10% Gibco certified FBS (Life Technologies, Grand Island, NY) plus antibiotic-antimycotic agents. Thereafter, the PBMCs, were stimulated either through the TCR as above to activate T cells [28] (TCR-CM), through the Toll-like Receptor 4 (TLR4) using 10 μg/mL lipopolysaccharide (LPS) to activate monocytes (LPS-CM), or left unstimulated (US-CM). After 48 hours, the resulting immune-cell-conditioned media (immune cell CM) were collected, centrifuged at 400*g*, passed through a 0.22-μm filter and stored frozen in multiple aliquots at -80°C. Prior to culturing with breast cancer cell lines, the CM were diluted 1:4 with the appropriate culture medium for each cell line. TGF-β1 (R&D Systems, Minneapolis, MN) was used at a concentration of 2 ng/mL in the appropriate media as a positive control for EMT induction.

### xCELLigence real-time growth, adhesion and migration assays

Changes in migratory and invasive capacity of SUM149 IBC cells, cell migration were quantified with an xCELLigence Real-Time Cell Analyzer using cell invasion and migration (CIM) plates (Acea Biosciences, Inc., San Diego, CA) in serum-free media using FBS as a chemoattractant for SUM149 cells. Growth and adhesion were measured using E-plates. The xCELLigence system detects changes in cell number, adhesion, and morphology that affect ionic impedance. This increased electrical impedance is normalized as a “cell index.” The CIM plate is essentially a Boyden chamber that measures increasing impedance on the lower surface of a semi-permeable membrane as cells migrate into the lower chamber [29]. To ensure that chemoattractant factors in the CM did not affect migration, 25% CM were placed in both the upper and lower chambers. The CM were diluted with serum-free IBC medium in the upper chamber, while the lower chamber used IBC medium supplemented with 10% FBS. Serum-free medium in the lower chamber served as a negative migration control. Cells were suspended in the appropriate medium and added to the upper chamber as per the manufacturer’s protocol. Impedance was measured on the receiving surface in the lower chamber as an indicator of cell migration and normalized to a cell index with RTCA software version 1.2.0.0909 (Roche Applied Science, Indianapolis, IN, under license from Acea Biosciences).

### Gene expression analysis by quantitative polymerase chain reaction

Following the treatment times as noted, samples were collected in Qiazol (Qiagen, Valencia, CA) and total RNA was extracted with the miRNeasy Mini Kit (Qiagen). cDNA was synthesized from 1 μg of RNA using the High-Capacity cDNA Reverse Transcription Kit (Life Technologies, Foster City, CA), as previously described [25]. Gene expression was quantified with RT-PCR using TaqMan gene expression assays (Life Technologies) listed in listed in S2 Table. Relative expression was quantified using the ΔΔCt method using glyceraldehyde 3-phosphate dehydrogenase (GAPDH) as the normalizer and the cells grown in IBC medium as the reference.

### Cell block and immunohistochemistry

Control cells (MCF-7 and MDA-MB-231) were grown on plastic in 2D cultures in the appropriate media as noted above, and SUM149 cells were exposed to CM for 48 hours. Cells were harvested following trypsinization and washed twice in PBS. Cytospins of 5x10^5^ cells were stained with Diff-Quik. Additionally, 2x10^7^ cells for each cell line were fixed in 10% formalin for 16 hours, pelleted by centrifugation, and embedded in paraffin, as previously described [30] to prepare cell blocks for sectioning and immunohistochemical analysis.

### Fluidigm qRT-PCR array

An extended panel of EMT-related mRNAs from multiple cell lines was prepared for gene expression using the BioMark 48.48 Dynamic Array and BioMark HD thermocycler (Fluidigm Corporation, San Francisco, CA) [31] after 14 cycles of pre-amplification using pooled TaqMan gene expression assays for specific target-amplification. Samples were analyzed using Real-Time PCR Analysis Software Version 2.0 (Fluidigm). Relative gene expression changes were calculated with the ΔΔCT method using *GAPDH* as the endogenous control and cells grown in the appropriate medium control as the normalizer for each cell line.

### Cytokine measurement

Small aliquots of CM were analyzed using Millipore Milliplex kits (EMD Millipore, Billerica, MA) according to the manufacturer’s protocol using a Luminex 100 system (Luminex, Austin, TX), as previously described [32]. Kits used included the human cytokine chemokine panel I, human bone panel, and TGF-β1.

### Signaling

To test the ability of conditioned media to induce TNF-α and IL-6 signaling pathways, phosphorylation of NF-kB and STAT3 were quantified. Cells were allowed to attach to tissue culture plastic and grow for 2 days. IBC medium was then exchanged for 25% CM. At specified time points, cells were harvested and placed in lysis medium provided as part of the Millipore MILLIPLEXMAP Signaling Magnetic Bead Kit—Cell Signaling Multiplex Assay (EMD Millipore, Billerica, MA) according to the manufacturer’s protocol and analyzed using Luminex 100.

## Results

### Patient blood shows correlation of immune activation and circulating tumor cells with EMT characteristics

The etiology of inflammatory breast cancer is poorly understood, but it has been postulated that an infectious process may precipitate or enhance the disease [33, 34]. Therefore, we hypothesized that the inflammatory environment created by activated immune cells can induce an EMT in breast cells. Since there is lymphatic and hematogenous dissemination of tumor to metastatic sites, isolation and interrogation of these cells in transit can give insight into the reasons for this highly aggressive tumor. We first examined blood from a small cohort of IBC patients for EMT-CTC.

We hypothesized that EMT could be induced by inflammatory processes initiated by activated immune cells. Therefore, we interrogated TCR-activated T-cells for their ability to synthesize TNF-α (TNT-T) (representative gating strategy shown in S1 Fig). Matched EMT-CTC and T-cell cytokine data were evaluable from 16 breast cancer patients, as shown in Table 1. Six patients had >225 TNF-T cells/ μL of blood and 4 of them had detectable EMT-CTC versus only 1 among 10 patients with <225 TNF-T cells/μL of blood” (Fisher’s exact test, p = 0.036). Although the number of patients is small, higher numbers of TNF-T cells were associated with a greater ability to detect EMT-CTC in breast cancer patients. These data suggest a relationship between activated immune cells and the metastatic potential of breast cancer cells.

**Table 1:** T cells producing TNF-α correlate with EMT-CTC in patients. PBMCs from breast cancer patients were stimulated overnight through the T-cell receptor with anti-CD3 and anti-CD28 antibodies and interrogated for intracellular TNF-α synthesis by multiparameter flow cytometry. Additionally, 5 mL of blood was depleted of CD45+ leukocytes to enrich for circulating tumor cell and interrogated for expression of EMT-related factors. There was a significant correlation between the detection of at least 1 EMT-related factor in the CTC-enriched fraction and presence of more than 225 CD3+ T-cells per μL of blood capable of producing TNF-α.

		TCR-activated CD3+ T Cells that produced TNF-α per μL blood of patient blood		Total
		*<225*	*>225*	
EMT CTC	*no EMT*	**9**	2	11
	*any EMT*	1	**4**	5
	Total	10	6	16

### Immune cell media induced cell growth, migration, and invasion as measured by live, label-free real-time cell analysis

**Fig 1:**
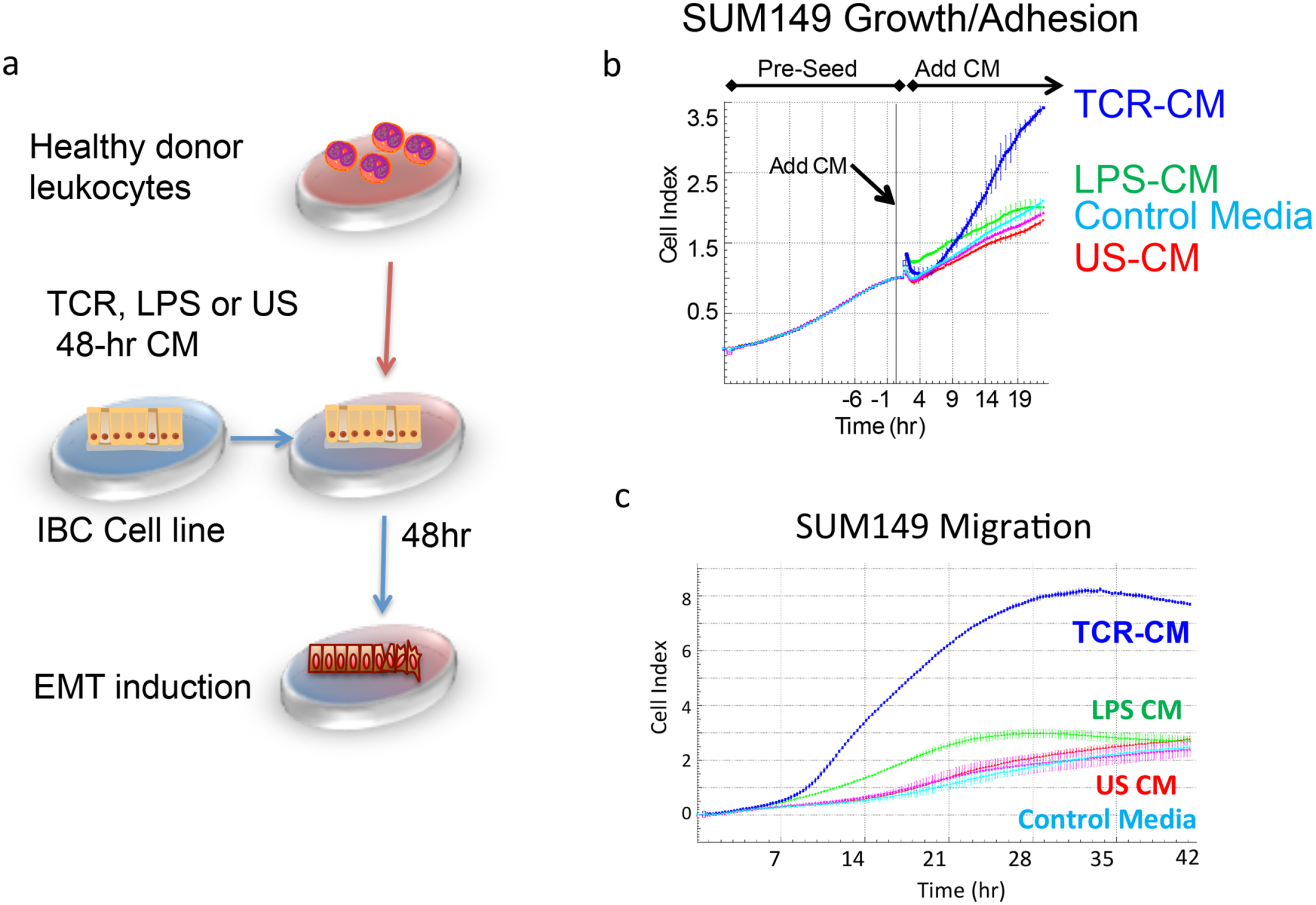
Immune conditioned media induces migration and adhesion in IBC cells. (a) Conditioned media (CM) was collected from healthy donor peripheral blood mononuclear cells stimulated for 48 hours with LPS (LPS-CM), through the T-cell receptor (TCR-CM) or left unstimulated (US-CM). CM were added to established SUM149 cultures at 25% of media volume and incubated an additional 48 hours prior assaying. (b) SUM149 were grown on an xCelligence E-plate and exposed to TCR-CM, LPS-CM and US-CM at time 0. Cell index was measured at 15-minute intervals. Robust changes are observed at 9 hours after treatment. (c) Migration towards fetal bovine serum (FBS) is enhanced by immune CM. TCR-CM (blue line) induces rapid migration of SUM149 cells. LPS-CM enhancement of migration (green line) is noted from 7 to ~36 hours, but is not significantly different from controls (US-CM, Media, no FBS; red, pink and light blue) at later time points.

Next, to test the ability of soluble factors secreted by activated immune cells to increase the metastatic potential of IBC cells, we established a culture system based on SUM149 IBC cells utilizing the CM created by activated immune cells of healthy donors. Using CM rather than co-culture avoided cell-cell contact, mitigating any cellular cytotoxicity (Fig 1a) and further allowed for the optimal use of media for each cell type, most specifically the use of hydrocodone in the IBC medium that would otherwise inhibit immune responses.

The TCR-CM was added to SUM149 cells and monitored in real-time using the Acea xCELLigence real-time cell analyzer. Within 10 hours of adding TCR-CM, the xCELLigence detected an increased cell index (Fig 1b), which is a measure of cell growth and adhesion derived from electrical impedance (see methods). Interestingly, although SUM149 cells treated with TCR-CM for 2 days clearly show decreased cell counts relative to media control or US-CM as assessed by manual traditional trypan blue exclusion assay (S2 Fig), the real-time analysis showed a marked increase in the cell index measurement (CIM). In fact, in 2 days, 8x10^3^ SUM149 cells treated with TCR-CM achieved a cell index about 2 times larger than that of 20x10^3^ SUM149 cells that were grown in IBC medium for 5 days; IBC medium SUM149 cells had a CIM of 2.54 after 22 hours of attachment and achieved a maximum CIM of 3.09 at 70 hours after seeding, while SUM149 cells treated with TCR-CM had a CIM of 1.20 after attachment that rapidly increased to 3.1 within 14 hours of treatment, and 6.07 within 43 hours of treatment. As the CIM by the xCELLigence is affected by cell morphology and adhesion in addition to cell growth, these data suggest that IBC cells that were exposed to TCR-CM increased adhesion to the either the extracellular matrix, neighboring cells, or both.

We therefore tested the ability of SUM149 cells exposed to immune cell CM to transverse a permeable membrane under chemotaxis towards a FBS gradient using xCELLigence cell invasion and migration (CIM) plates. SUM149 cells exposed to TCR-CM showed a very rapid increase in the cell index, suggesting an induced migratory capacity (Fig 1b). Similar migration patterns were observed when the plates were precoated with 15% Matrigel and when cells were pretreated with immune cell CM for 48 hours and loaded into the chambers in equal numbers (data not shown). Combined, these data show that the changes induced in IBC cells by soluble immune factors paradoxically increase both adhesion and migration reminiscent of a transient EMT.

### Immune Conditioned media induced EMT-like changes in IBC cells

**Fig 2:**
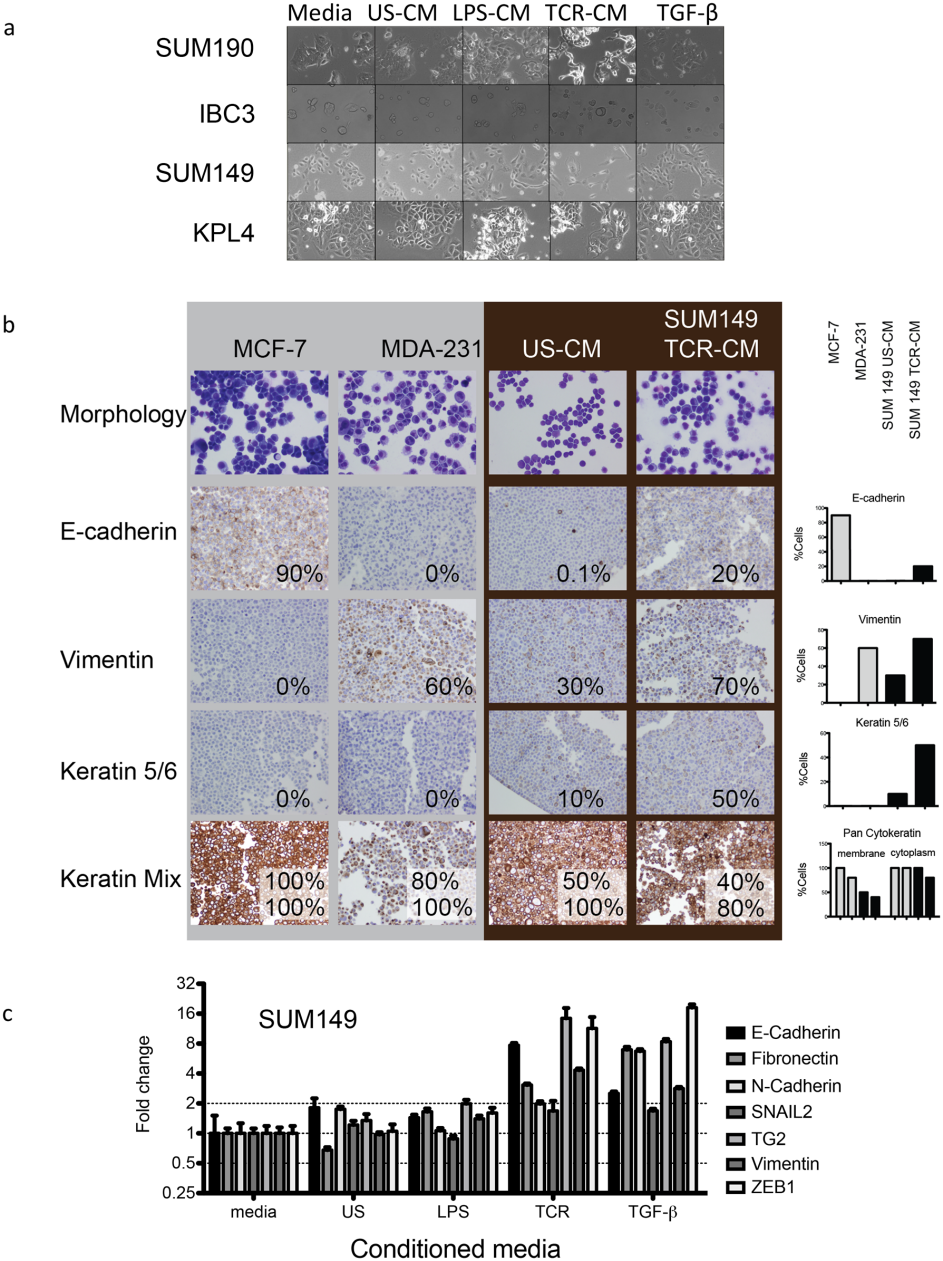
Conditioned media from activated healthy donor PBMC induces EMT in IBC. (a) Morphological changes visualized by bright field in IBC cell lines consistent with stress and EMT were observed following 48-hour incubation with CM. (b) Immunohistochemical stains of paraffin cell blocks: the percentage of positive cells is listed in each image and shown in the bar graph at right. Pancytokeratin expression is shown for both percent of cells with membrane localization (top number) and cytoplasmic localization (bottom number). MCF-7 control cells show a characteristic epithelial phenotype with high E-cadherin, low vimentin, low keratin 5/6 expression and strong membrane and cytoplasmic localization of cytokeratins, MDA-231 control cells are mostly mesenchymal with low E-cadherin, high vimentin and decreased cytokeratins. Following exposure to TCR-CM, SUM149 cells show increased expression of E-cadherin, vimentin, keratin 5/6 staining and decreased pan cytokeratin staining. (c) Expression levels of EMT-related transcription factors SNAIL, ZEB1 and TG2 were quantified by Taq-Man qRT-PCR. TCR-CM and to a lesser extent LPS-CM, induced large increases in ZEB1 and TG2.

To test the hypothesis that soluble factors secreted by activated immune cells can induce EMT in IBC cells, CM were prepared from activated immune cells and added to established 2D cultures of IBC cell lines SUM149, SUM190, IBC3 and KPL4. Cells were grown 2 days in the presence of CM prior to assaying (see Fig 1a for schema). Morphological examination under bright field revealed that cells cultured with TCR-CM and to a lesser extent, cells cultured with LPS-CM, exhibited a mesenchymal or stressed morphology with elongated projections consistent with EMT (Fig 2a).

EMT-like changes were also observed in protein expression and localization in SUM149 following incubation with TCR-CM when compared to reference epithelial (MCF-7) and mesenchymal (MDA-MB-231) cell lines (Fig 2b). Following treatment, paraffin-embedded cell blocks were stained by immunohistochemistry for pathological review. In a standard IBC culture medium, SUM149 cells had an epithelial expression pattern similar to luminal MCF-7 cells. After incubation with TCR-CM, SUM149 cells have a mesenchymal phenotype more similar to the basal/mesenchymal MDA-MB-231[35]. Consistent with the luminal-type phenotype, MCF-7 cells expressed high E-cadherin and keratin mix (stained with an anti-CK antibody cocktail) but lacked expression of vimentin or cytokeratins (CK)-5/6. In contrast, MDA-MB-231 cells stained for vimentin in more than 50% of cells, lacked E-cadherin expression, and keratin mix reactivity was localized mostly in the cytoplasm (with a punctate staining suggestive of Golgi localization), consistent with the basal/mesenchymal-type phenotype.

SUM149 cells treated with TCR-CM had increased expression of vimentin and E-cadherin. Furthermore, we found an increase of CK-5/6 expression typical of basal-like breast cancer cells. Finally, CK localization to both the membrane and cytoplasm decreased following TCR-CM treatment, consistent with a loss of epithelial phenotype. Together, the increased staining of vimentin and CK-5/6 in conjunction with the decreased pan-CK suggest that factors secreted by activated PBMC induce cellular changes consistent with an epithelial to mesenchymal transition.

A small, targeted analysis of gene expression in SUM149 cells by q-PCR showed that TCR-CM induced at least a two-fold increased expression of fibronectin (*FN1*), vimentin (*VIM*), N-cadherin (*CDH2*), *TGM2*, and *ZEB1*, and slight increase in snail (*SNAI1*) (Fig 2c). Interestingly, although the increased expression of these EMT-regulating genes is typically associated with a decreased expression of E-cadherin, SUM149 cells showed a substantial increase in E-cadherin expression in response to TCR-CM (Fig 2b and 2c). This paradoxical increase in E-cadherin is consistent both with the increased adhesion observed in SUM149 cells in the real-time cell analysis (Fig 1) and the unique E-cadherin expression pattern in tumor samples from IBC patients [36] where E-cadherin in maintained even in metastatic sites[37].

Together, these data show that after exposure to immune cell CM, SUM149 IBC cells lose epithelial characteristics and gain mesenchymal characteristics suggesting an epithelial to mesenchymal transition with a unique retention of E-cadherin.

### Immune cell CM induced a broad EMT profile IBC and non-IBC breast cancer cell lines

To test immune induction of EMT in additional genetic backgrounds and against a broader array of pathways, immune cell CM was added to a panel of breast cancer cell lines described in S1 Table. In addition to SUM-149, three additional IBC cell lines and 5 non-IBC cell lines were evaluated using the same pool of CM. Morphological changes imaged under bright field are shown in S3 Fig. Cells exposed to immune cell CM were generally less dense and appeared spindly and stressed.

**Fig 3:**
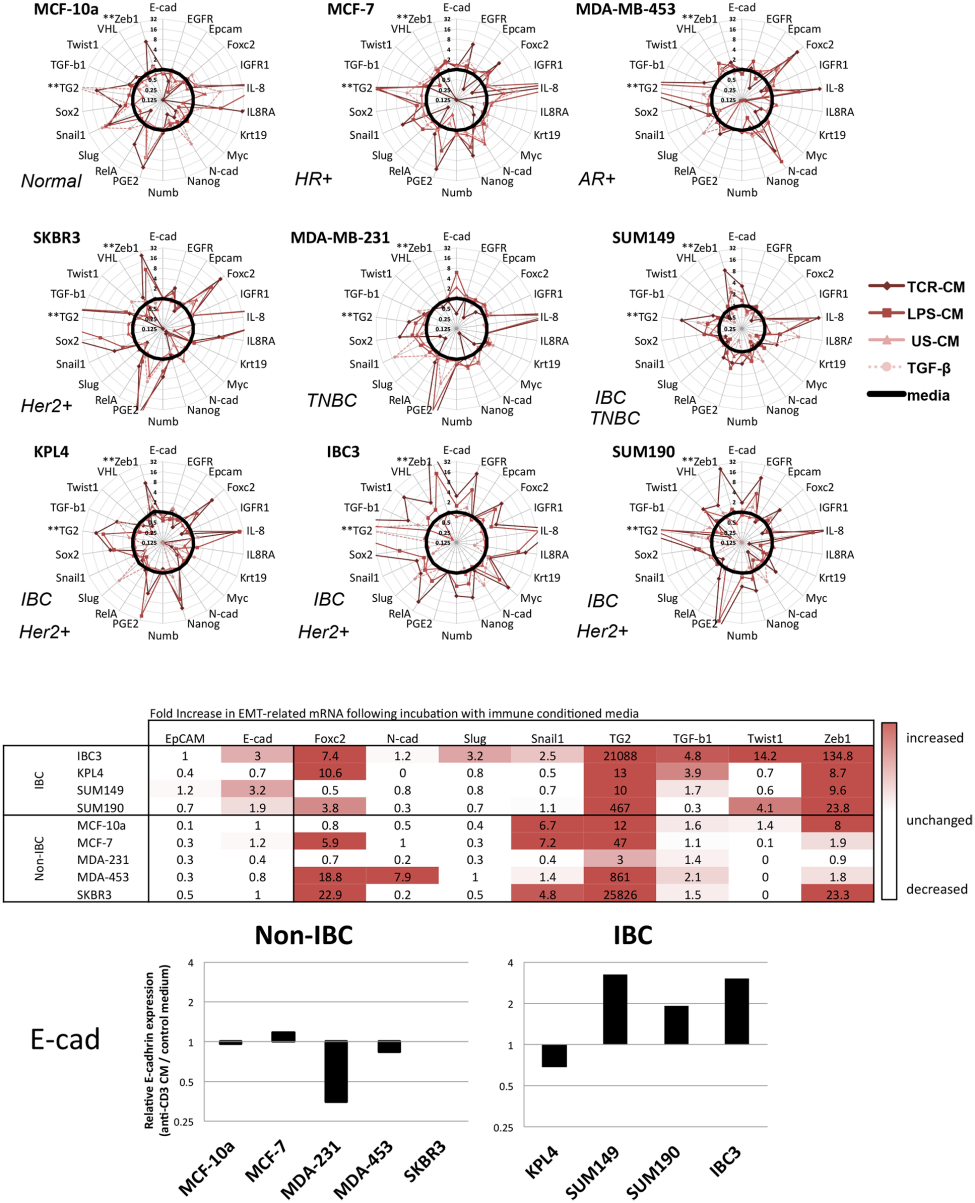
Analysis of multiple cell lines by Fluidigm. Breast cancer cell lines were incubated with immune CM for 48 hours and mRNA was analyzed by qRT-PCR. Media control appears as a solid black line at 1 in the center of each plot, points falling outside the circle represent increased relative expression and lines inside are decreased. TCR-CM anti-CD3 induced EMT-related factors to varying degrees in all cell lines. Following treatment with TCR-CM, IBC cell lines with the exception of KPL4, had increased expression of E-cadherin.

A panel of 24 selected genes was analyzed for each condition using Fluidigm integrated fluidic circuit chips (Fig 3). Most cell lines showed increased expression of EMT-regulating genes following treatment with immune cell CM. Changes in response to LPS-CM were generally attenuated compared to TCR-CM, possibly due to the smaller relative number of monocytes than T cells in PBMC. *ZEB1* and *TGM2* were the most frequently, and highly induced EMT-related factors, along with inflammatory factors prostaglandin E synthase 2 (*PTGES2*) and IL-8 (*CXCL8*). Consistent with induction of EMT in epithelial cells, epithelial cell adhesion molecule (EpCAM) expression decreased with TCR-CM treatment in every cell line except SUM149. However, in the other IBC cell lines, although EpCAM expression was reduced, this down-regulation was attenuated compared to the non-IBC cell lines (p = 0.0197). Likewise, although the non-IBC cell lines showed no change in E-cadherin expression at this time point, 3 of the 4 IBC cell lines (SUM149, SUM190, and IBC3, but not KPL4) showed the paradoxical increase in E-cadherin expression noted previously (p = 0.0411). While these data confirm that immune induction of EMT is common among a range of breast cancer subtypes, IBC cells had an abnormal response characterized by a shift toward EMT expression concurrent with maintained or increased E-cadherin expression.

### Profile of cytokines secreted by human activated PBMC CM

**Fig 4:**
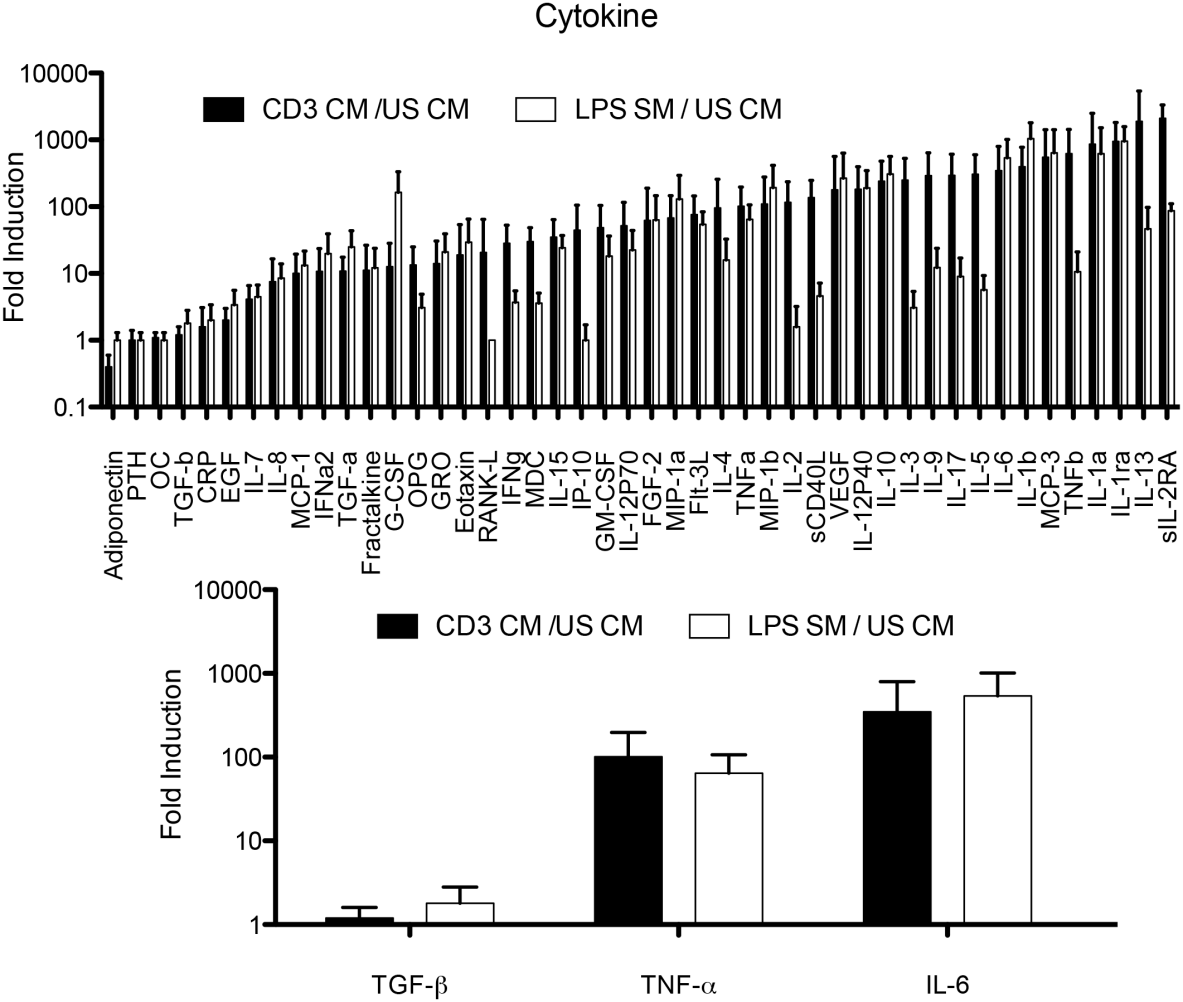
Characterization of conditioned media from activated healthy donor PBMC. Cytokine concentrations in the CM supernatants were measured by Luminex multiplex array in 5 representative normal donors. a) Relative expression of 46 cytokines, chemokines and growth factors; b) relative expression of TNF-α, TGF-β and IL-6 in LPS-CM, TCR-CM relative to US-CM (solid bars).

As it was previously shown that inflammatory cytokines (TNF-α, IL-6, TGF-β) could promote EMT, we confirmed the presence of these molecules in immune cell CM. We characterized the TCR-CM, LPS-CM, and US-CM from 5 healthy donors using Luminex multiplex bead assay. The TCR-CM contained at least a 100-fold increase in the following factors: interferon (IFN)-γ, IL-1α, -1β, -2, -3, -5, -6, -9, -10, -12p40, -13, -17, monocyte chemotactic protein (MCP)-3, macrophage inflammatory protein-1 beta (MIP-1β, soluble CD40 ligand (sCD40L), soluble IL-2 receptor alpha (sIL-2Rα), TNF-α, TNF-β and vascular endothelial growth factor (VEGF). In the LPS CM, a 100-fold increase or greater was likewise observed in the following cytokines: granulocyte colony stimulating factor (G-CSF), IL-1α, -β, -6, -10, -12p40, IL-1 receptor antagonist (IL-1RA), monocyte chemotactic protein 3 (MCP-3), MIP-1α, -1β, and VEGF (Fig 4a). We found high levels of inflammatory cytokines that regulate EMT (i.e.: TNF-α and IL-6). Specifically, TCR-CM, had an average 101-fold increase for TNF-α; an average 347-fold increase for IL-6 and a modest average 1.6-fold increase for TGF-β (Fig 4b). A similar pattern was obtained from LPS-CM (Fig 4b). These results indicated that upon activation, both the lymphocyte and monocyte components of PBMC produce cytokines that have the potential to induce EMT.

### Neutralizing the effects of TNF-α, TGF-β, and IL-6 reversed EMT phenotype induced by immune conditioned media

**Fig 5:**
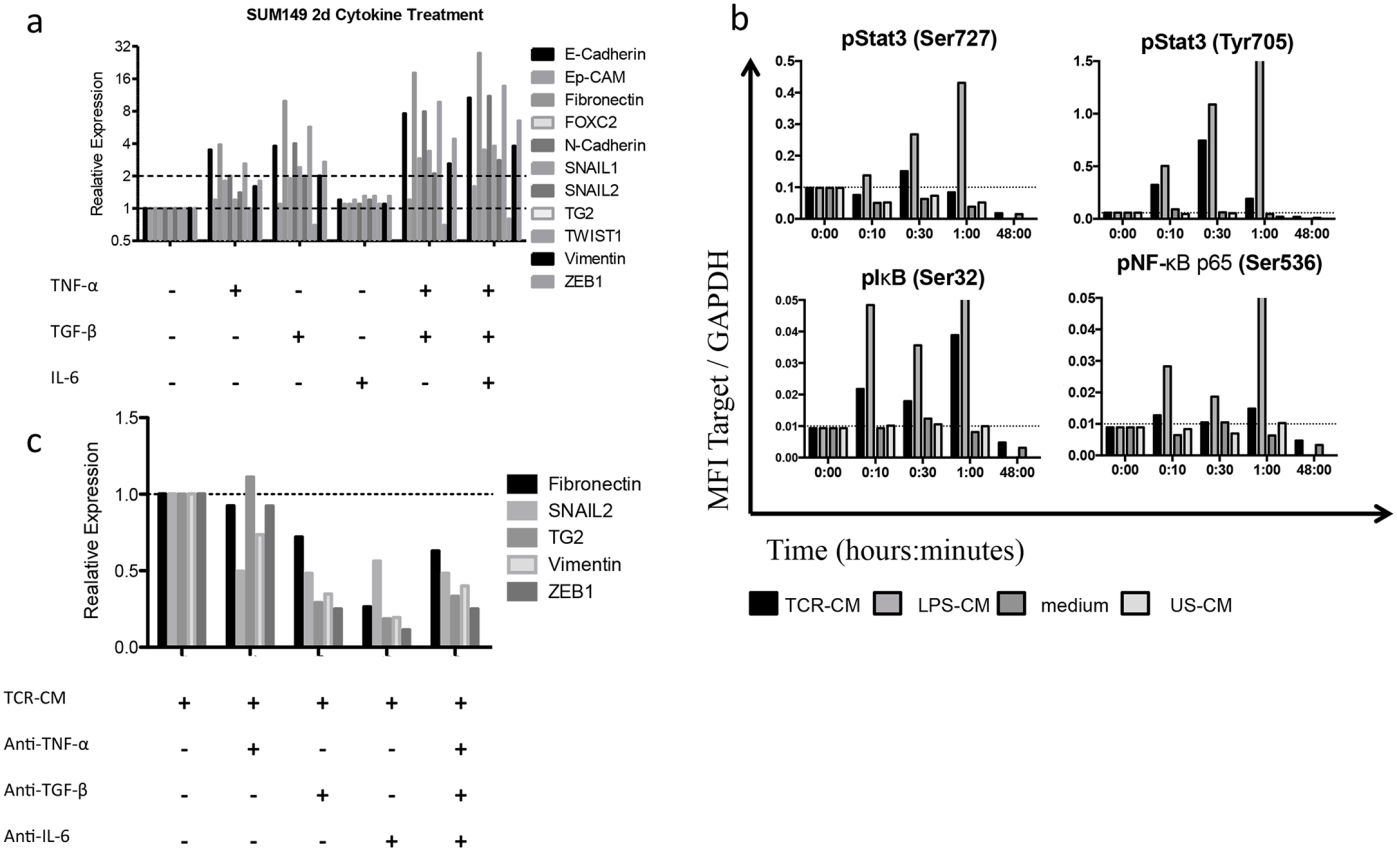
TNF-α, TGF-β and IL-6 induce EMT in SUM149. a) TNF-α, TGF-β and IL-6 were added to established SUM149 cultures and assayed for EMT-inducing transcription factors (EMT-TF). All three cytokines act additively to induce EMT-TF. B) TCR-CM induced STAT3 and NF-κB signaling in SUM149 cells as quantified by phospho-specific antibodies using Luminex multiplex beads. C) Neutralizing antibodies against TNF-α, TGF-β and IL-6 were added to TCR-CM prior to incubating with SUM149 cells. Compared to TCR-CM without neutralization, EMT-TF expression was reduced.

As TNF-α, TGF-β1 and IL-6 have been shown to induce EMT in other systems, we evaluated the individual and combinatorial effects of these three cytokines in the induction of EMT in IBC cells. Addition of human recombinant (hr) TNF-α, (hrTNF-α, hrTGF-β, or hrIL-6 synergistically induced EMT in SUM149 cultures as measured by the qPCR expression of EMT-related factors (Fig 5a). As TNF-α and IL-6 signal through NF-κB and Stat3, respectively, we hypothesized that incubation with TCR-CM would increase activation of these pathways. After analysis with the Millipore MultiplexMAP bead assay, whole cell lysates of SUM149 cells showed increased Stat3 and NF-κB phosphorylation following incubation with TCR-CM (Fig 5b), suggesting that this CM is capable of inducing these pathways in tumor cells. Therefore, we hypothesized that blocking TNF-α, TGF-β, and IL-6 would mitigate the induction of EMT by immune activation. To test this hypothesis, TCR-CM was pre-absorbed with neutralizing antibodies to each of TNF-α, TGF-β1 and IL-6 prior to incubation with SUM149 cells in culture. Compared with TCR-CM, the pre-absorbed CM showed a reduction in the expression of E-cadherin (CDH1), *EPCAM*, *FN1*, *CDH2*, *SNAI2*, *TGM2*, *VIM*, and *ZEB1* in SUM149 IBC cells (Fig 5c). These data suggest that TNF-α, TGF-β1 and IL-6 are partially responsible for the immune-induced changes observed in IBC cells.

## Discussion

In this study, we have shown that soluble factors secreted by activated PBMC of healthy donors including TNF-α, IL-6 and TGF-β can induce a paradoxical co-expression of EMT signaling and E-cadherin overexpression in IBC cells. Blocking these factors can partially prevent the development of this EMT phenotype. This paradoxical increase in migratory behavior and concomitant expression of the adhesion molecule E-cadherin may account for the tight adherence of IBC cell clusters into tumor emboli that obstruct lymphatics and lead to the “inflammatory” symptoms of IBC. These data implicate immune cell activation in mediating aspects of the IBC phenotype in vitro, and highlight new targets for further clinical investigation.

Induction of EMT-like features by immune cells has been shown in other systems. For example, monocytes derived factors have been implicated in IBC models [5, 6] and macrophage TNF-α, IL-6, and IL-1β can induce TGF-β and EMT in MCF-7 cells [38]. Here, our LPS stimulation recapitulates these data and that soluble factors produced by TCR-activated T-cells are also able to induce EMT. Likewise, Soria et al. showed that TNF-α and IL-1β, expressed in over 80% of patients with relapsed breast cancer, induce hallmarks of EMT *in vitro* but show concurrent increases in both migration and adhesion [39] similar to the data presented here.

Inflammation and the immune response have long been viewed as a delicate balance that have the ability to promote a durable tumor regression or promote tumor progression [40]. A Th2-polarized T cell response, characterized traditionally by the cytokine IL-4 but also TNF-α and IL-6[41], has been shown to support tumor growth [42]. In contrast, a Th1 immune response is typically associated with tumor control. The TCR-activation used here induces polyclonal stimulation of T cells that activates both Th1 and Th2 polarized cells with little bias [43]. Therefore, soluble factors from multiple T-cell subsets are present in the TCR-CM. However, the apparent importance of TNF-α, IL-6, and TGF-β in EMT induction suggest that a Th2, and not Th1 polarization, contributes to this process. This is consistent with microarray data from the World IBC Consortium that recently showed that a Th1 gene signature was associated with attainment of a pathological complete response in IBC patients [44] suggesting that tipping the immune response towards Th1 and away from TH2 and the induction of EMT is beneficial in IBC.

Twist is one of the primary transcription factors responsible for driving EMT, yet we did not observe a consistent change in Twist expression in this study. However, Twist induces invasion, in part, through upregulation of platelet-derived growth factor receptor (PDGF-R) [45, 46]. As PDGF-R expression is constitutively high in IBC [47], Twist may be dispensable for invasive properties in IBC. Furthermore, under transient exposure to TGF-β1, Snail1 actively represses early Twist1 expression at two days, prior to Twist induction [48], consistent with the data shown here. Therefore, the lack of increased Twist expression in response to soluble inflammatory factors in our data can be consistent with EMT induction.

The combination of increased invasion and increased adhesion observed with the xCELLigence platform and suggested by the PCR data is perplexing but not unprecedented. The increased cell index has also been reported in the benign prostate hyperplasia cell line BPH-1 in response to TGF-β1 [49] using the same xCELLigence platform used here. The authors argued that although TGF-β1 has anti-proliferative effects, the induction of EMT promotes cell spreading that increases cell index.

No immune competent *in vivo* model of IBC has been published making it difficult to study the interaction of immune and IBC tumor cells. In this study, blood samples from patients suggested a correlation between TNF-α production by T-cells and the presence of EMT in CTC. The co-culture system employed here allowed testing of directional interactions from immune cells to tumor cells and showed that soluble factors from activated immune cells induce EMT in tumor cells.

This immune-induced EMT had striking differences from other breast cancer models. EMT and metastatic progression are typically associated with loss of E-cadherin; indeed, even in the short time period in this study, the non-IBC cell lines maintain or decrease E-cadherin. In stark contrast, IBC cell lines increased expression of E-cadherin following exposure to soluble factors secreted by activated T cells. As such, the expression of E-cadherin in response to inflammatory signaling may contribute to the unique presentation of IBC.

Changes in population phenotype can be attributed to either the induction of new pathways (*i*.*e*. EMT) or the selection of pre-existing rare populations. In support of the latter, inflammatory factors (such as TNF-α) induce death in the majority of cells and select for resistant stem cells that are characterized by EMT phenotypes [50]. Accordingly, our data confirm that the TCR-CM greatly reduces cell counts relative to controls, although we cannot confirm if the mechanism is due to a cytostatic effect or a combination of reduced proliferation and cell death. However, the kinetic data from the xCELLigence platform suggest selection of resistant cells is unlikely to account for the increase in EMT phenotype as the cell index increases far faster than stem cells would be able to repopulate a nascent niche evacuated by dead differentiated cells. Therefore, the observed increase in EMT phenotype is likely a result of EMT induction rather than selection of resistant stem-cell like cells.

The data here showed that spiking IL-6 into the media had little effect on the induction of EMT, yet depleting IL-6 from CM drastically reduced the observed EMT at the 2-day time point. It is possible that IL-6 plays a minor role inducing EMT but has a strong role in maintaining the EMT-like or stem-like state [51]. The data here suggest that multiple pathways need to be blocked to prevent EMT induction, but preventing the maintenance of this state may offer a better target.

These results reinforce the importance of inflammation in cancer progression. Indeed, anti-inflammatory regimens have been related to decreased risk of breast cancer in most studies [52]. Recently, a retrospective chart review of IBC patients found that lipophilic statins, which are taken as cholesterol-lowering agents but also have potent anti-inflammatory effects [53], were associated with increased progression-free survival [54] but not overall survival, suggesting that these anti-inflammatory drugs are ineffective once metastases have formed [55]. This is consistent with our observations that inflammatory factors induce EMT and suggests that inflammation can induce metastatic transformation offering a prime target for therapeutic targeting. Furthermore, as induction of EMT can promote immune evasion [56], reducing these inflammatory effects may promote better tumor control by the immune system.

In summary, we have shown that immune factors can induce phenotypic, morphological, and functional changes in breast cancer cells that are associated with EMT. Unique to IBC model cells, E-cadherin fails to respond to the EMT program consistent with clinical observations of maintained E-cadherin in IBC patients. It is possible that similar inflammatory conditions *in vivo* may support both the rapid metastasis and tight tumor emboli that are characteristic of IBC and that targeted anti-inflammatory therapy may be advantageous in this patient population.

## Supporting Information

S1 Fig Gating strategy for TNF-α synthesis by T cells. Archived PBMC from breast cancer patients were stimulated overnight through the T-cell receptor with plate-bound anti-CD3 and soluble anti-CD28. The total number of CD3+CD4+ and CD3+CD4- (assumed to be CD8+) T cells synthesizing TNF-α were enumerated by flow cytometry. The percentage was back calculated based on cell surface phenotypes obtained from fresh whole blood from the same sample to determine the number of T cells per ml of blood capable of synthesizing TNF-α. Representative sample shown.
(PDF)

S2 Fig Pretreatment with immune-cell conditioned media decreases cell recovery. Cells were seeded and allowed to attach for 2 days prior to adding immune conditioned media. After an additional 2 days of culture with immune conditioned media, cells were counted manually using trypan blue exclusion. Cell counts are plotted and the cell viability is noted to the right of each respective bar. Cells incubated with TCR-CM had had fewer cells than control cells although viability was not affected.
(PDF)

S3 Fig Morphological changes induces in breast cancer cell lines by immune conditioned media. Immune-cell-conditioned media were added to established cultures of 10 breast cancer cell lines and cultured for 2 days. Bright-field images are shown. Most cell lines show a shift towards a mesenchymal-like phenotype with increased projections and fewer, looser cell clusters.
(PDF)

S4 Fig Conditioned media from activated healthy donor PBMC induces EMT in IBC. LPS-CM, omitted in fig 2, is shown. The morphological changes observed in LPS-CM conditioned breast cancer cells are not robust, likely due to the relatively small effect LPS-CM has due to the smaller number of cells in PBMC that respond to LPS (primarily monocytes which typically constitute 10% of PBMC).
(PDF)

S1 Table Breast cancer cell lines. The breast cancer type, sources, and growth media of cell lines used are outlined.
(PDF)

S2 Table RT-PCR TaqMan assays TaqMan gene expression assays were used for RT-PCR as listed. (PDF)
