# Supplementary material for: Inflammation Mediated Metastasis: Immune Induced Epithelial-To-Mesenchymal Transition in Inflammatory Breast Cancer Cells
Source: PLoS One. 2015 Jul 24;10(7):e0132710. doi: 10.1371/journal.pone.0132710 (PMC4514595; doi:10.1371/journal.pone.0132710)
Supplement: S1 Fig — Archived PBMC from breast cancer patients were stimulated overnight through the T-cell receptor with plate-bound anti-CD3 and soluble anti-CD28. The total number of CD3+CD4+ and CD3+CD4- (assumed to be CD8+) T cells synthesizing TNF-α were enumerated by flow cytometry. The percentage was back calculated based on cell surface phenotypes obtained from fresh whole blood from the same sample to determine the number of T cells per ml of blood capable of synthesizing TNF-α. Representative sample shown. (PDF) [file pone.0132710.s001.pdf]

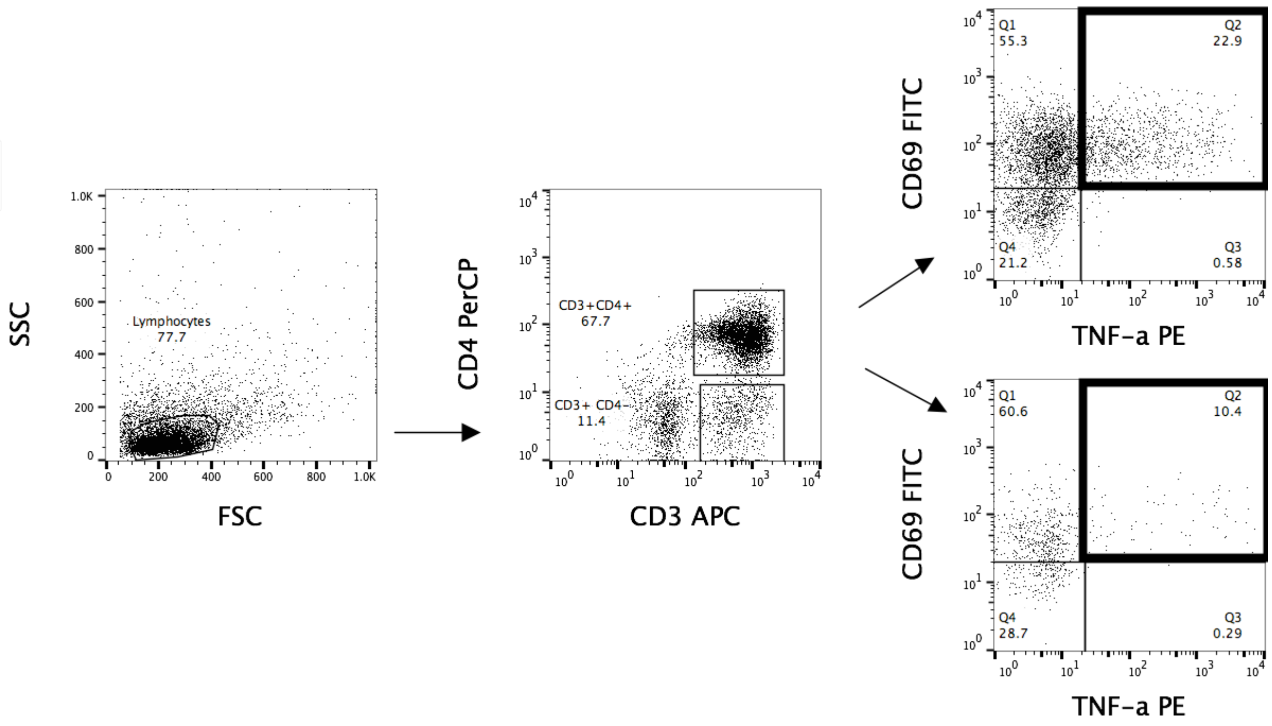

Supplementary Figure 1. Gating strategy for TNF-α synthesis by T cells. Archived PBMC from breast cancer patients were stimulated overnight through the T-cell receptor with plate-bound anti-CD3 and soluble anti-CD28. The total number of CD3+CD4+ and CD3+CD4- (assumed to be CD8+) T cells synthesizing TNF-α were enumerated by flow cytometry. The percentage was back calculated based on cell surface phenotypes obtained from fresh whole blood from the same sample to determine the number of T cells per ml of blood capable of synthesizing TNF-α. Represnetaive sample shown.
