# Supplementary material for: Inflammation Mediated Metastasis: Immune Induced Epithelial-To-Mesenchymal Transition in Inflammatory Breast Cancer Cells
Source: PLoS One. 2015 Jul 24;10(7):e0132710. doi: 10.1371/journal.pone.0132710 (PMC4514595; doi:10.1371/journal.pone.0132710)
Supplement: S2 Fig — Cells were seeded and allowed to attach for 2 days prior to adding immune conditioned media. After an additional 2 days of culture with immune conditioned media, cells were counted manually using trypan blue exclusion. Cell counts are plotted and the cell viability is noted to the right of each respective bar. Cells incubated with TCR-CM had had fewer cells than control cells although viability was not affected. (PDF) [file pone.0132710.s002.pdf]

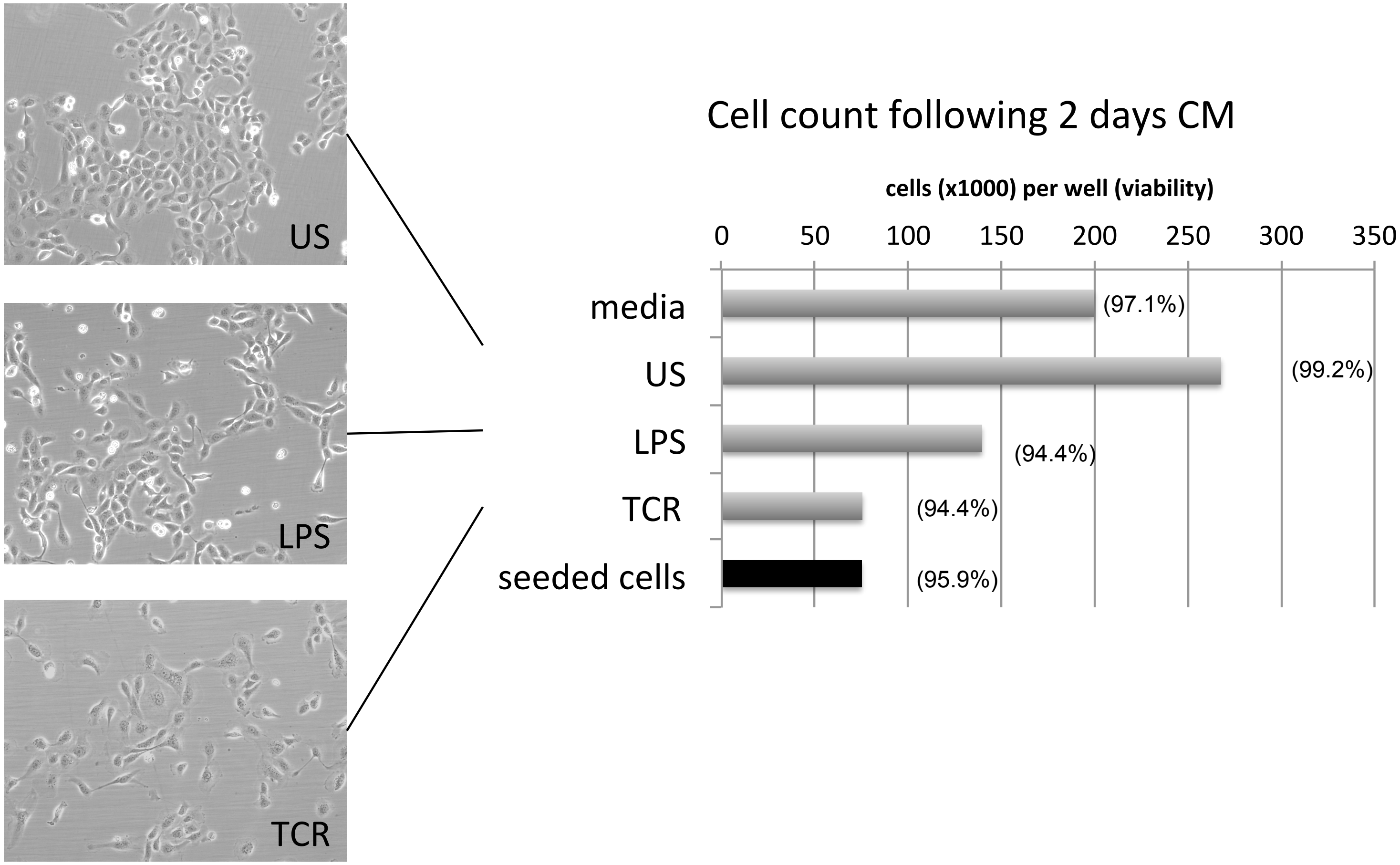

Supplementary Figure S2. Pretreatment with immune-cell conditioned media decreases cell recovery. Cells were seeded and allowed to attach for 2 days prior to adding immune conditioned media. After an additional 2 days of culture with immune conditioned media, cells were counted manually using trypan blue exclusion. Cell counts are plotted and the cell viability is noted to the right of each respective bar. Cells incubated with TCR-CM had had fewer cells than control cells although viability was not affected.
