# Supplementary material for: Inflammation Mediated Metastasis: Immune Induced Epithelial-To-Mesenchymal Transition in Inflammatory Breast Cancer Cells
Source: PLoS One. 2015 Jul 24;10(7):e0132710. doi: 10.1371/journal.pone.0132710 (PMC4514595; doi:10.1371/journal.pone.0132710)
Supplement: S3 Fig — Immune-cell-conditioned media were added to established cultures of 10 breast cancer cell lines and cultured for 2 days. Bright-field images are shown. Most cell lines show a shift towards a mesenchymal-like phenotype with increased projections and fewer, looser cell clusters. (PDF) [file pone.0132710.s003.pdf]

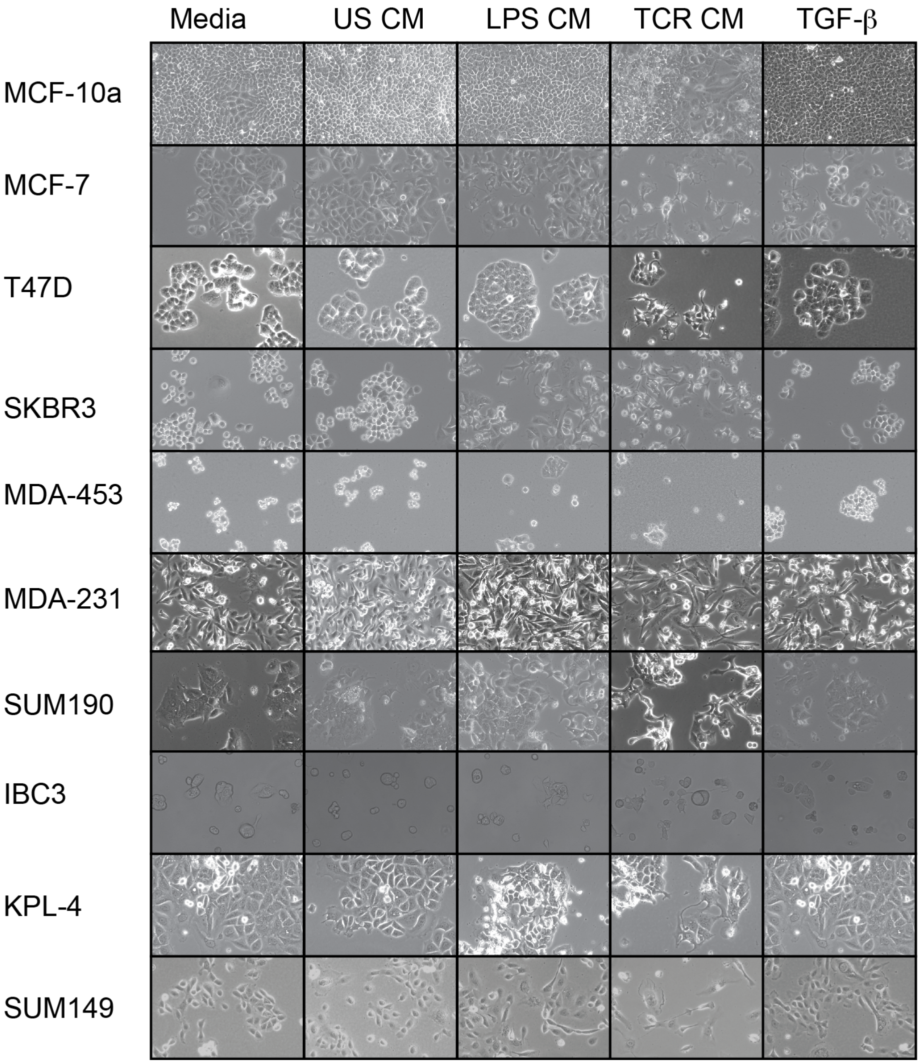

Supplementary Figure 3. Morphological changes induces in breast cancer cell lines by immune conditioned media. Immune-cell-conditioned media were added to established cultures of 10 breast cancer cell lines and cultured for 2 days. Bright-field images are shown. Most cell lines show a shift towards a mesenchymal-like phenotype with increased projections and fewer, looser cell clusters.
