# Supplementary material for: Inflammation Mediated Metastasis: Immune Induced Epithelial-To-Mesenchymal Transition in Inflammatory Breast Cancer Cells
Source: PLoS One. 2015 Jul 24;10(7):e0132710. doi: 10.1371/journal.pone.0132710 (PMC4514595; doi:10.1371/journal.pone.0132710)
Supplement: S4 Fig — LPS-CM, omitted in fig 2, is shown. The morphological changes observed in LPS-CM conditioned breast cancer cells are not robust, likely due to the relatively small effect LPS-CM has due to the smaller number of cells in PBMC that respond to LPS (primarily monocytes which typically constitute 10% of PBMC). (PDF) [file pone.0132710.s004.pdf]

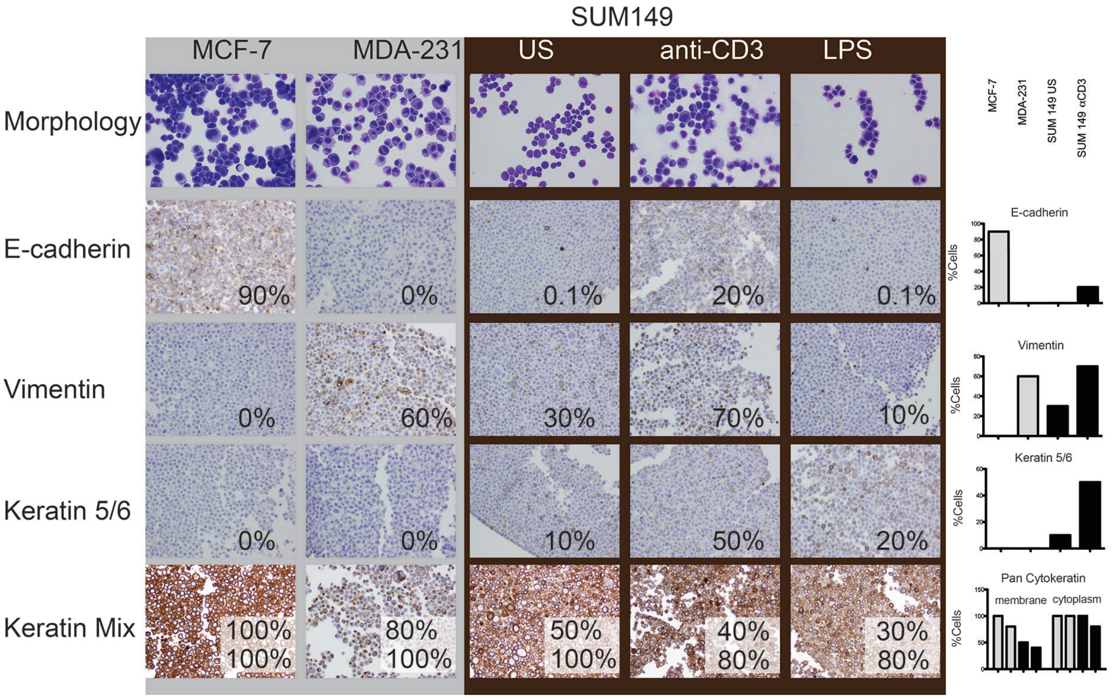

Supplemental Figure S4. Conditioned media from activated healthy donor PBMC induces EMT in IBC. LPS-CM, omitted in figure 2, is shown. The morphological changes observed in LPS-CM conditioned breast cancer cells are not robust, likely due to the relatively small effect LPS-CM has due to the smaller number of cells in PBMC that respond to LPS (primarily monocytes which typically constitute 10% of PBMC).
