# Supplementary material for: Inflammation Mediated Metastasis: Immune Induced Epithelial-To-Mesenchymal Transition in Inflammatory Breast Cancer Cells
Source: PLoS One. 2015 Jul 24;10(7):e0132710. doi: 10.1371/journal.pone.0132710 (PMC4514595; doi:10.1371/journal.pone.0132710)
Supplement: S1 Table — The breast cancer type, sources, and growth media of cell lines used are outlined. (PDF) [file pone.0132710.s005.pdf]

Supplementary Table 1 Breast cancer cell lines

Cell Line	IBC	Breast Cancer Type	Source	Growth Medium
SUM149PT	IBC	basal	Stephen Ethier (Asterand, Detroit, MI)	Ham's F12 5% FBS HI
KPL4	IBC	HER2-amplified	Dr. Junichi Kurebayashi (Kawasaki Medical School, Kurashiki, Japan)	Ham's F12 5% FBS HI
IBC-3	IBC	HER2-amplified	Dr. Wendy Woodward (MD Anderson Cancer Center, Houston, TX)	Ham's F12 5% FBS HI
SUM190PT	IBC	HER2-amplified	Stephen Ethier (Asterand, Detroit, MI)	Ham's F12 5% FBS HI
MCF-7	non-IBC	luminal	ATCC	DMEM/F12 10% FBS
MFC-10A	non-IBC	non-tumorigenic	ATCC	DMEM/F12 5% Horse Serum, Cholera Toxin, EHI
MDA-MB-231	non-IBC	basal, highly mesenchymal	ATCC	DMEM/F12 10% FBS
MDA-MB-453	non-IBC	androgen receptor-positive	ATCC	DMEM/F12 10% FBS
SKBR3	non-IBC	HER2-amplified	ATCC	DMEM/F12 10% FBS

H = hydrocortisone, I = insulin, E = epidermal growth factor
