# Supplementary material for: Inflammation Mediated Metastasis: Immune Induced Epithelial-To-Mesenchymal Transition in Inflammatory Breast Cancer Cells
Source: PLoS One. 2015 Jul 24;10(7):e0132710. doi: 10.1371/journal.pone.0132710 (PMC4514595; doi:10.1371/journal.pone.0132710)
Supplement: S2 Table — (PDF) [file pone.0132710.s006.pdf]

Supplementary Table 2 RT-PCR TaqMan® assays

Alias	Gene Symbol	Assay ID	Gene Name
18S	18S	Hs99999901_s1	Eukaryotic 18S rRNA
*GAPDH	GAPDH	Hs02758991_g1	glyceraldehyde-3-phosphate dehydrogenase
*E-cadherin	CDH1	Hs01023894_m1	cadherin 1; E-cadherin (epithelial)
EGFR	EGFR	Hs01076078_m1	epidermal growth factor receptor
*EpCAM	EPCAM	Hs00158980_m1	epithelial cell adhesion molecule
*Fibronectin	FN1	Hs00365052_m1	fibronectin 1
*FOXC2	FOXC2	Hs00270951_s1	forkhead box C2 (MFH-1; mesenchyme forkhead 1)
IGF1R	IGF1R	Hs00609566_m1	insulin-like growth factor 1 receptor
IL-8	IL8	Hs00174103_m1	interleukin 8
KRT19	KRT19	Hs00761767_s1	keratin 19
MYC	MYC	Hs00153408_m1	v-myc avian myelocytomatosis viral oncogene homolog
*N-cadherin	CDH2	Hs00983056_m1	N-cadherin (neuronal)
Nanog	NANOG	Hs04260366_g1	Nanog homeobox
Numb	NUMB	Hs01105433_m1	numb homolog (Drosophila)
PTGES2	PGE2	Hs00228159_m1	prostaglandin E synthase 2
RELA	RELA	Hs00153294_m1	v-rel avian reticuloendotheliosis viral oncogene homolog A
*Snail1	SNAI1	Hs00195591_m1	snail family zinc finger 1
*Slug	SNAI2	Hs00950344_m1	snail family zinc finger 2
Sox2	SOX2	Hs01053049_s1	SRY (sex determining region Y)-box 2
TGF-B1	TGFB1	Hs00998133_m1	transforming growth factor; beta 1
*TG2	TGM2	Hs00190278_m1	transglutaminase 2
*TWIST1	TWIST1	Hs00361186_m1	twist basic helix-loop-helix transcription factor 1
VHL	VHL	Hs03046964_s1	von Hippel-Lindau tumor suppressor; E3 ubiquitin protein ligase
*Vimentin	VIM	Hs00185584_m1	vimentin
*Zeb1	ZEB1	Hs00232783_m1	zinc finger E-box binding homeobox 1

*Assays run in limited IBC tests
